# Nutritional, Physiochemical, and Antioxidative Characteristics of Shortcake Biscuits Enriched with *Tenebrio molitor* Flour

**DOI:** 10.3390/molecules25235629

**Published:** 2020-11-30

**Authors:** Ewelina Zielińska, Urszula Pankiewicz

**Affiliations:** Department of Analysis and Evaluation of Food Quality, University of Life Sciences in Lublin, Skromna Str. 8, 20-704 Lublin, Poland; ewelina.zielinska@up.lublin.pl

**Keywords:** edible insects, entomophagy, shortcake biscuits with insects, mealworm, novel protein

## Abstract

Edible insects, due to their high nutritional value, are a good choice for traditional food supplementation. The effects of partial replacement of wheat flour and butter with mealworm flour (*Tenebrio molitor*) on the quality attributes of shortcake biscuits were studied. The approximate composition was analyzed, along with the physical properties and color. Moreover, the antioxidant properties, starch digestibility, and glycemic index were determined in vitro. The protein and ash contents in biscuits supplemented with mealworm flour increased, while the carbohydrates content decreased. The increasing insect flour substitution decreased the lightness (L*) and yellowness (b*) but increased the redness (a*), total color difference (ΔE), and browning index (BI). The spread factor for the sample with the highest proportion of mealworm flour was significantly higher than the other biscuits. Furthermore, higher additions of mealworm flour increased the antioxidant activity of the biscuits and contributed to an increase in the content of slowly digested starch, with a decrease in the content of rapidly digested starch. Therefore, the results of the research are promising and indicate the possibility of using edible insects to enrich food by increasing the nutritional and health-promoting values.

## 1. Introduction

Entomophagy is commonly practiced by 2 billion people mainly in Asia, Africa, and South America [1]. In recent years, insect consumption has also been promoted in areas where it is not a tradition, such as Europe and North America, because of the high nutritional value and low environmental impact associated with eating insects [2]. Edible insects are known to be good sources of proteins, lipids, certain vitamins, and minerals, such as calcium, iron, or zinc. Importantly, insects are good sources of essential amino acids and polyunsaturated fatty acids [3].

Despite the advantages of using insects in human nutrition, the barrier to wider consumption is negative consumer perception. Insects in Western countries have several bad associations, namely with dirtiness, poverty, and diseases [4]. Visual impressions are very important because they are the consumer’s first chance to form an opinion of the product; therefore, promoting the consumption of whole insects is not popular in Western societies. Sensory aspects of food are very important for consumer acceptance, so it is a good idea to design insect-based foods with minimal negative sensory characteristics [5]. Moreover, it has been confirmed that the addition of insects to food products in an invisible way allows for the acceptance of these products [6,7]. Insect-based foods started with the addition of insects into familiar food products such as bread [8,9], frankfurters [10], pastes [11,12], bakery products [13,14], and snacks bars [15] or cereal-based snacks [16]. In particular, cereal-based foods such as bread, biscuits, bakery products, and pasta are very popular and highly accepted worldwide, and thus research on how to enrich them with insect flours would be a good starting point. Additionally, insect flours could be sources of protein in gluten-free products when substitutes for gluten protein are sought [17]. Based on previous research, we can generally conclude that the addition of insects to enriched food products increased the contents of protein and ash and decreased the carbohydrate content [18]. However, the addition of insects can also enrich products with valuable fatty acids. Edible insects are certainly rich in unsaturated fatty acids, with levels comparable to those of poultry and fish, however containing more polyunsaturated fatty acids (PUFAs). One of the insects that is abundant in unsaturated fatty acids is the mealworm (*Tenebrio molitor*). The unsaturated fatty acid content in *T. molitor* amounts to 74.64% [3,19]. There is a growing number of papers discussing the use of insect flour, many of which focus on mealworms. For example, supplementation of maize tortillas with mealworm powder contributed to an increase in the contents of protein and essential amino acids [20]. In turn, the presence of mealworm flour in wheat bread increased the dough’s stability and tenacity, as well as the crumb density [9]. This type of supplementation also affected the nutritional value of the bread—it increased the protein, fat, and ash contents [14]. For cookies supplemented with mealworm flour, the moisture, carbohydrate, protein, fat, ash, and mineral contents were higher compared with control cookies [21]. Other changes in products supplemented with mealworm flour include visual aspects. In one study, the lightness and yellowness of the muffins decreased with an increase in the mealworm powder concentration, however the redness increased [22]. An attempt was made to replace pork meat with mealworms in Frankfurters. The sausages formulated with a combination of 40% pork meat and 10% mealworm were similar in terms of cooking loss, emulsion stability, protein solubility, and overall acceptability to regular control frankfurters, maintaining the overall quality [10].

Furthermore, edible insects, in addition to their high nutritional value, have nutraceutical properties, such as strong antioxidant properties [23]. Our previous studies showed that after the in vitro digestion and absorption process, edible insects, including *T. molitor*, still have high antiradical activity [24,25,26]. Therefore, their addition to food products can increase the nutritional and pro-health value. Moreover, among the edible insects that are popular in Europe, *T. molitor* is characterized by its reasonably high fat content, with an appropriate fatty acid profile [3]. Based on the composition of mealworm meal, we can conclude that it should be used in the supplementation of traditional foods rich in flour and fat. An example is a shortcake, which is the basis of many food products (not only confectionery products, but also baking tarts and salty pastries). As such, in our research, we decided to replace some of the flour and butter in shortcake biscuits with ground mealworms. We examine the extent to which replacing some of the flour and butter with insect flour could influence the nutritional, physiochemical, and antioxidative properties of food products.

## 2. Results and Discussion

### 2.1. Physical Properties

The physical properties of the biscuits were determined by weight, diameter, thickness, spread factor, and apparent density. The physical properties of the biscuits are shown in Table 1. The biscuit weights ranged from 10.88 g to 13.41 g, with sample modification 1 (M1) presenting the lowest weight value (10.88 g) as compared to the control (C) (12.37 g). This decrease may be associated with the weaker water-binding capacity of mealworm flour than wheat flour, which may be responsible for moisture loss during the baking process, resulting in the lower weight of the biscuit. Moreover, in modification 1, as a result of the proportion of ingredients applied to the dough, the smallest amount of water was introduced in this batch. The smallest amounts of butter and flour were also used, with these ingredients being replaced by mealworm flour, which contains less moisture. The diameters of the biscuits were not significantly different (*p* < 0.05) and varied from 4.94 mm to 4.98 mm, whereas for the thickness, only sample M1 was significantly lower than the others (6.89 mm) (*p* < 0.05), which is correlated with the low weight of this sample. Therefore, the spread factor for this sample was significantly higher than the other biscuits (7.18) (*p* < 0.05). Undoubtedly, this was a result of the largest amount of mealworm flour being added to this batch. The spread ratio is a measure of cookie quality. A higher spread ratio is desirable for better cookies, which indicates higher product performance [27]. On this basis, it can be concluded that a higher addition of insect flour may increase the product’s yield. Similar results were noted for cookies prepared from amaranth flour—the obtained spread factor was 7.95 [28]. The spread ratios of the other samples did not differ significantly from each other (*p* < 0.05). For the studied biscuits, we also calculated the apparent density, which was significantly higher for biscuits with higher amounts of mealworm flour (M1 and modification 2 (M2)) than the others. Although there is not much research on insect supplementation of bakery products, cookies are a very popular matrix for assessing supplementation with other functional additives. Certain functional additives, such as the insect flour used in this study, cause similar changes in the physical properties of bakery products. For example, cookies enriched with dephytinized oat bran [29] and novel cookies with a sclerotium of edible mushroom [30] had higher spread ratios.

### 2.2. Nutrient Composition

The approximate compositions of the studied biscuits and mealworm flour are reported in Table 2. Mealworms are a good source of compounds of high nutritional value, such as proteins, lipids, and particularly polyunsaturated fatty acids and minerals, such as iron, magnesium, and zinc [3]. Edible insects are known as high-protein food products, and the protein content of the mealworms was determined to be 54.6% of the dry weight (d.w.). Furthermore, insects are rich in minerals—the ash content was determined to be 3.89% d.w. The substitution of wheat flour with mealworm flour changed the nutritional value of the products. The protein content of the biscuits with the addition of mealworm flour ranged from 10.82% to 13.52% of dry weight. This effect was expected because mealworm flour is the richest in protein among all ingredients in the recipe, so the increase in its content causes a proportional increase in protein content in the end product. In turn, the fat content was at a similar level in all biscuits (*p* < 0.05). In this case, the fat content that was removed from the dough in the form of butter was almost completely replaced in the form of fat from mealworms, and the estimated fat contents introduced into the dough from the ingredients in all modification batches were similar. Biscuits with the highest amounts of mealworm flour (M1 and M2) were found to be the richest in ash, with levels equivalent to the microelement contents (0.63 and 0.7, respectively). Considering possible ash content deviations, sample M3 was characterized by having the lowest value due to the having the lowest amount of added mealworm flour. Generally, there was a progressive increase in the protein and ash contents, with a decrease in the carbohydrates content as the concentration of mealworm flour increased. In turn, the moisture content decreased as the mealworm flour concentration increased in the biscuits. The water contained in the dough was derived only from water contained in the ingredients used for its production—no additional water was used. This proportional change in the moisture content was, therefore, caused by the replacement of the flour and butter with mealworm flour, which has a lower moisture content than these ingredients, meaning the water content of the dough was also reduced. The nutritional compositions of the products are important, however the moisture content is a significant quality factor affecting preservation, packaging, and transport convenience [31]. Although the proportions of protein and carbohydrates had changed, no effects on the energy values of the tested biscuits were found (*p* < 0.05). This is because protein and carbohydrates have the same conversion factor values as energy values [32]. A difference in energy value could only be caused by a significant difference in fat content.

### 2.3. Color Measurements

**Figure 1:**
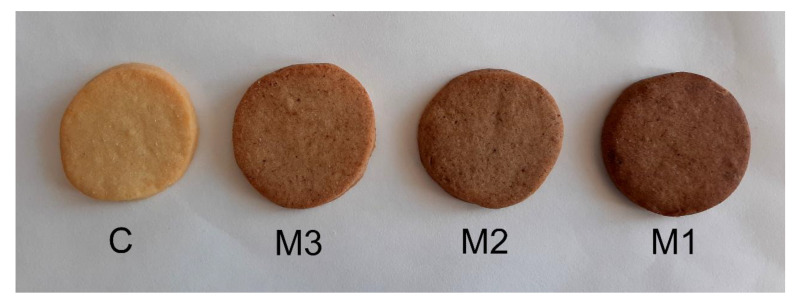
Shortcake biscuits prepared with different amounts of mealworm flour and wheat flour. C—control sample; M1—modification 1; M2—modification 2; M3—modification 3.

The surface colors of the shortcake biscuits in terms of the L*, a*, b*, ΔE, and browning index (BI) values can be found in Table 3. The appearances of the biscuits are shown in Figure 1. Increasing the amount of insect flour decreased the lightness (L*) and yellowness (b*) while increasing the redness (a*), total color difference (ΔE), and browning index. Such results were expected, because mealworm flour is darker than the wheat flour used in these biscuits, hence supplementation with mealworm flour will give products a darker color, because usually the color of a baked product is directly dependent on the colors of the raw materials used. On the other hand, the protein content in mealworm flour was higher than in wheat flour, resulting in a higher degree of Maillard reaction with increased surface redness [33]. Similar results for color darkening were observed by researchers for muffins enriched with mealworm [22] and cricket powder [18], cookies enriched with mealworm powder [21], bread supplemented with insect flour [8,14], and pasta enriched with cricket powder [17]. The highest color difference in the control samples was seen in sample M1, which contained the greatest amount of mealworm flour. As Pauter et al. [18] suggested, consumers tend to see darker bakery products as healthier and containing more fiber or whole grains. Therefore, this color change may increase consumer interest in this type of biscuit.

### 2.4. Antioxidant Properties

**Figure 2:**
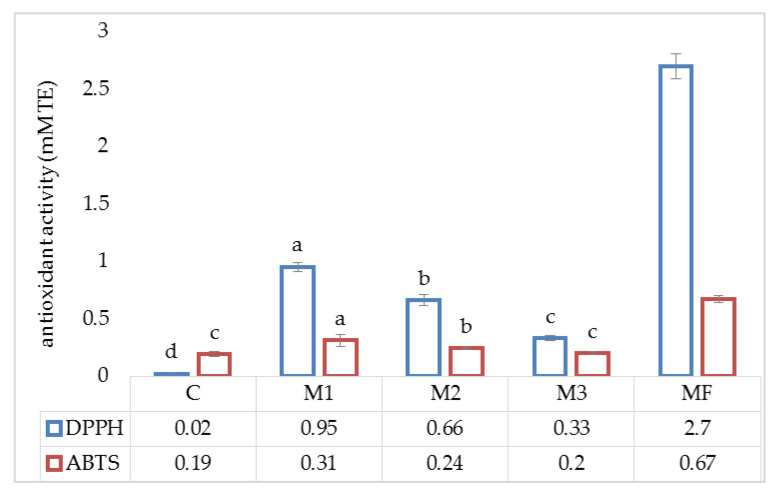
Antioxidant properties of biscuits. C—control sample; M1—modification 1; M2—modification 2; M3—modification 3; MF—mealworm flour. Values followed by a different superscript in a column differ significantly (*p* < 0.05).

Measuring the antioxidant capacity of food products is of increasing interest because it can provide a wide variety of information on factors such as the oxidation resistance, the quantitative contribution of antioxidants, or the antioxidant effects that can occur in the body at the time of consumption. The antioxidant activity of the sample extracts was evaluated by assessing the ability of the extracts to inhibit 2,2-diphenyl-1-picrylhydrazyl (DPPH^•^) and 2,2′-azino-bis(3-ethylbenzothiazoline-6-sulfonic acid (ABTS^•+^). The antioxidant properties of the mealworm flour and the biscuits prepared from it are shown in Figure 2. The highest antiradical activity values against both ABTS^•+^ and DPPH^•^ were observed for the mealworm flour (0.67 and 2.70 mM TE, respectively). Navarro del Hierro et al. [34] studied the DPPH^•^ scavenging activity of mealworm extracts and also confirmed their strong antioxidant properties. As expected, the partial substitution of wheat flour with mealworm flour significantly (*p* < 0.05) increased the free radical scavenging capacity, as reflected by DPPH^•^ and ABTS^•+^ scavenging activity values. Therefore, the antioxidant activity levels of the biscuits increases as the concentration of mealworm flour in the recipe increased. Higher free radical scavenging activity was observed for DPPH^•^ than ABTS^•+^. Food fortification has gained increased interest among consumers. Many products are enriched to increase their antioxidant potential. For example, cookies produced with 5% to 20% camu camu (*Myrciaria dubia*) coproduct powder as a replacement for wheat flour had higher antioxidant potential than control cookies [35]. In turn, cookies supplemented with flaxseed in amounts of 5% and 30% exhibited DPPH radical scavenging activity values of 7.93% and 12.25% as compared to 5.5% for the control [31]. Food enrichment with unconventional protein sources is becoming increasingly popular. For example, the incorporation of microalgae in amounts ranging from 2% to 6% into cookies led to a significant increase in the antioxidant capacity [36]. As unconventional sources of protein, insects may, therefore, have good potential to enrich food and improve its nutraceutical value. The antioxidant activity of bread enriched with cricket flour as measured by the DPPH and ABTS scavenging activity showed was significantly higher than standard dough [37]. Many bioactive compounds have been identified in insects (e.g., chitins, polyphenols, antioxidant enzymes, peptides, proteins, etc.) [23,24,26,38,39]. As high-protein products, insects are, therefore, potential sources of bioactive proteins and peptides. Numerous amino acid sequences from insects have been identified, which have been associated with in vitro bioactive properties [23,26,40,41,42]. Proteins and peptides are also involved in the antioxidant properties of insects [25,26,43], and depending on the species of edible insects these proteins might change the DPPH^•^ and ABTS^•+^ radical scavenging activity. Such changes might be depend on the molecular weight of protein or peptide in question, as well as the amino acid composition [44]. Moreover, based on the high activity of DPPH^•^ in terms of radical scavenging, the obtained results suggest that mealworm proteins contain amino acids or peptides that act as electron donors and can react with free radicals to transform them into more stable compounds.

It has been confirmed that during in vitro digestion of the mealworm, peptides with strong antioxidant properties are released. Their activity has been confirmed based on the activity of the synthesized peptide sequences. The identified peptides also showed higher activity against DPPH^•^ than ABTS^•+^. The following peptides were identified in the hydrolysates of *T. molitor*: NYVADGLG from raw insect cuticle protein, AAAPVAVAK from boiled insect cuticle protein, YDDGSYKPH from baked insect ADFb protein, and AGDDAPR from isolated protein [26]. Moreover, the results of the presented study show that the heat treatment used for the insects increased the antiradical activity of the peptide fractions, with baking yielding particularly good results [25].

### 2.5. Rapidly and Slowly Digested Starch Contents and In Vitro Glycemic Index (GI) Values

Rapidly digested starch (RDS) is defined as the part of the starch that is digested within 20 min of food intake and which causes a rapid increase in blood glucose levels. Slowly digested starch (SDS) is the part of the starch that is completely digested in the small intestine, but at a slower rate than RDS, i.e., within 20–120 min of ingestion [45].

Based on the obtained results, it can be concluded that the addition of flour from mealworm affected the changes in rapidly and slowly digested starch contents (Table 4). All samples were found to have more rapidly than slowly digested starch, but the control and modification 3 (M3) samples contained three times more RDS than SDS, whereas the M1 sample contained only about 1.8 times more RDS than SDS. Generally, samples M1 and M2, which contained the highest amounts of mealworm flour, had significantly lower contents of RDS and higher contents of SDS than control, which is desirable for consumers. The dietary benefits attributed to SDS are associated with a slower postprandial rise in blood glucose and glycemia maintenance for longer periods compared to RDS, which results in a rapid rise and then a rapid fall in blood glucose, often to below the initial value due to the increased insulin levels. The prolonged absorption of glucose after consumption of products rich in SDS inhibits the release of free fatty acids from adipose tissue. Reducing their inflow to the liver promotes the faster removal of glucose from the cardiovascular system and consequently leads to a decrease in the serum concentration. These features of SDS give products rich in this component low glycemic index values [46].

The effect of the mealworm flour addition on starch digestibility in biscuits has been documented. The digestibility of starch present in food, especially when subjected to different types of heat treatment, is influenced by the other ingredients present in the food. The other ingredients can include proteins; interactions between proteins and starch are crucial [47]. The addition of edible insects as high-protein foods to bakery products can, therefore, lead increase interactions and also increase the content of slowly digested starch.

An in vitro starch hydrolysis method was used in this study to estimate the metabolic glycemic response to food products. The in vitro glycemic index values of the biscuits are shown in Table 4. The addition of mealworm flour to the biscuits did not affect the in vitro glycemic index values. So far, no effects of supplementation with edible insects on starch composition, digestibility, and in vitro glycemic index values have been shown in the literature. Further studies on these issues are, therefore, needed to increase the knowledge of food supplementation with edible insects.

## 3. Materials and Methods

### 3.1. Chemicals, Reagents, and Biscuit Ingredients

Trolox (6-hydroxy-2,5,7,8-tetramethylchroman-2-carboxylic acid), ABTS (2,2′-azino-bis(3-ethylbenzothiazoline-6-sulfonic acid), DPPH (2,2-diphenyl-1-picrylhydrazyl), dinitrosalicylic acid, pepsin from porcine gastric mucosa (cat no. 10080, 250 U/Mg), pancreatin from porcine pancreas (cat no. P1750), amyloglucosidase (cat no. 10115, 70 U/Mg), and invertase (cat no. I4504, 300 U/Mg) were purchased from Sigma-Aldrich Company, Ltd. (St Louis, MO, USA). The D-glucose assay kit (GOPOD format) was obtained from Megazyme International (Wicklow, Ireland). All other chemicals used were of analytical grade.

Wheat flour (type 500), sugar, eggs, and butter (80% fat, including 55% saturated fatty acids) were purchased from a local market. The *Tenebrio molitor* mealworms (Linnaeus, Coleoptera: Tenebrionidae) (larvae) were obtained from a local breeder (Lublin, Poland). The insects were fasted for two days to empty their digestive tract, then they were frozen for 24 h at −18 °C and lyophilized. Afterward, the insects were ground in a laboratory grinder (IKA A11 basic) to obtain flour. In order to obtain a uniform particle size, the flour was passed through a 20 mesh sieve, then the flour was analyzed (protein: 54.6 ± 2.2% d.w.; fat: 29.47 ± 1.95% d.w.; ash: 3.89 ± 0.8% d.w.; carbohydrates: 12.04 ± 0.96% d.w.; moisture: 4.72 ± 0.12%; energetic value: 532 ± 4.2 kcal/100g d.w. or 2223 ± 13.5 kJ/100g d.w). All analyses were performed within two weeks of the preparation of the flour, during which time it was stored in an airtight container at −18 °C. All chemicals and reagents used were of analytical grade.

### 3.2. Shortcake Biscuit Preparation

The biscuits were prepared as three variants with different amounts of added insect flour, which partially replaced the wheat flour and butter. Control biscuits were prepared without the addition of insects. The shortcake recipe contained commonly used ingredients, including wheat flour (300 g), butter (150 g), eggs (60 g), and sugar (70 g) [48]. The cold ingredients were then kneaded manually into dough, cooled, then sheeted manually on a dough sheeter to a uniform thickness of 0.5 cm, cut into round shapes measuring 5 cm in diameter, and baked at 180 °C for 25 min in a preheated oven. The biscuits were placed randomly to minimize the impact of their placement on the tray on their characteristics during baking. The biscuits were then allowed to cool at room temperature and subjected to physical and color measurements. Next, the biscuits were frozen at −18 °C so that additional analyses could be performed within two weeks. The quantities of ingredients for each biscuit variant are presented in Table 5.

### 3.3. Physical Properties

Fifteen biscuits were randomly selected from each batch and were analyzed in terms of weight, thickness, and diameter. The spread ratio was calculated by finding the ratio of a biscuit’s diameter versus its thickness [30]. The apparent density was calculated by evaluating the weight-to-volume ratio [49].

### 3.4. Nutrient Composition

The insect flour and biscuit samples were assessed for their moisture, ash, fat, and protein contents (N×6.25) by employing standard methods of analysis [50]. The carbohydrate content was determined by the following formula: 100 − (weight in grams (protein + fat + ash) in 100 g of the dry weight of edible insects). The conversion method was used for the determination of the nutritional value [33].

### 3.5. Color Measurements

Fifteen biscuits were randomly selected from each batch and color measurements were performed in triplicate. The color of the biscuits was measured using an EnviSense colorimeter NH310 (EnviSense, Lublin, Poland). Color differences were recorded in the CIE L*a*b* scale in terms of lightness (L*) and color (a*—redness; b*—yellowness). Additionally, the total color difference (ΔE) was calculated using the following formula:

(1) $$\Delta E=\sqrt{\Delta L^{*2}+\Delta a^{*2}+\Delta b^{*2}}$$

where ΔL*, Δa*, and Δb* are differences in the L*, a*, and b* values, respectively, between the reference sample and the test sample.

The browning index (BI, Equations (2) and (3)) was calculated using the measured L*, a*, and b* values as follows [51]:

(2) $$\mathrm{BI}=\;\frac{100\left(x-0.31\right)}{0.17}$$

where x:

(3) $$x=\;\frac{\left(a^{*}+1.75L^{*}\right)}{\left(5.645L^{*}+a^{*}-0.012b^{*}\right)}$$

### 3.6. Antioxidant Properties

#### 3.6.1. Extraction of Bioactive Compounds

The biscuits samples (1 g) were ground in a laboratory grinder and shaken with 10 mL of 4:1 ethanol/water (*v/v*) for 120 min in a laboratory shaker. Next, the samples were centrifuged at 3000 g for 10 min. The supernatant was stored at −18 °C until the next day for further analysis.

#### 3.6.2. DPPH Radical Scavenging Activity

The DPPH assay was estimated according to Brand-Williams, Cuvelier, and Berset [52], with slight modification. A 0.1 mL volume of the sample was mixed with 0.9 mL of a 6 µM solution of DPPH^•^ in 75% methanol. The absorbance was measured after 3 min of reaction at 515 nM. Here, 75% methanol was used as a blank. The scavenging effect was calculated using the formula:

(4) Scavenging activity (%) = [1 − (A sample/A control)] × 100

where A sample is the absorbance of the mixture of sample and DPPH^•^; A control is the absorbance of the control (DPPH^•^ solution).

The results were expressed as Trolox equivalent antioxidant activity (TEAC) values (mM Trolox).

#### 3.6.3. ABTS Radical Scavenging Activity

The ABTS assay was determined according to Re et al. [53] with slight modifications regarding the quantities of the antioxidant solution. The radical solution was prepared with ABTS and potassium persulfate, diluted in water to a final concentration of 2.45 mM, and left in the dark for 16 h to allow for radical development. The solution was diluted to reach the absorbance measures around 0.7 at 734 nM. Then, 2.95 mL of the ABTS^•+^ solution was mixed with 0.05 mL of each sample. The absorbance was measured after 3 min of the reaction at 734 nM. Deionized water was used as a blank. The scavenging effect was calculated according to the equation:

(5) Scavenging activity (%) = [1 − (A sample/A control)] × 100

where A sample is the absorbance of the mixture of sample and ABTS^•+^; A control is the absorbance of the control (ABTS^•+^ solution).

The results were expressed as Trolox equivalent antioxidant activity (TEAC) values (mM Trolox).

### 3.7. In Vitro Digestion of Biscuits

The in vitro digestibility of the starch was determined according to the method of Monro et al. [45] with slight modifications. Briefly, 1 g of the sample was combined with 30 mL of water and 0.8mL of 1M HCl to reach a pH of 2.5, then 1mL of 10% pepsin solution in 0.05M HCl was added. The reaction was carried out for 30 min at 37 °C with stirring (130 rpm) to complete gastric digestion. Next, 2mL of 1M NaHCO_3_ and 5mL of 0.1 M phosphate buffer (pH 6) were added, followed by 4.6 mg amyloglucosidase and 5mL of 2.5% pancreatin in 0.1 M phosphate buffer (pH 6) to start the small intestinal phase. The tubes were filled with distilled water to a volume of 55 mL. Digesta aliquots measuring 1.0 mL were removed after the gastric phase and at 20, 30, 60, 90, 120, and 180 min from the start of amylolysis, then were each subsequently added to 4mL absolute ethanol in a tube and mixed.

### 3.8. Rapidly and Slowly Digested Starch Contents

The rapidly digested starch (RDS) and slowly digested starch (SDS) levels were determined by measuring reducing sugars released during in vitro digestion of biscuits according to the procedure described by Soong, Tan, Leong, and Henry [54]. Briefly, sugars released during in vitro digestion were evaluated as monosaccharides using a dinitrosalicylic acid (DNS) colorimetric method with slight modifications. To complete the depolymerization into monosaccharides, 50 µL of the supernatant was mixed with 0.25 mL of acetate buffer containing 0.4% invertase and 1% amyloglucosidase. Incubation was carried out at room temperature for 30 min. Next, 0.75 mL of DNS mixture containing 0.5 mg/mL glucose, 4 M NaOH, and DNS reagent at a 1:1:5 ratio was added and heated for 15 min at 95–100 °C, cooled, and then diluted with 4 mL distilled water. The absorbance of the samples and standards was read at 530 nM against the blank.

The amount of sugars released was calculated in mg of glucose/g of each sample using the absorbance values. The rapidly digested starch (RDS) was evaluated as the amount of reducing sugars determined in the sample aliquot after 20 min from the start of pancreatic digestion, whereas slowly digested starch (SDS) was calculated as the difference between the amount of reducing sugars measured at 120 min and RDS.

### 3.9. In Vitro Glycemic Index (GI)

The in vitro GI of the biscuits was determined by evaluation of the in vitro starch digestibility according to the method described by Reis and Abu-Ghannam [55] with slight modifications, using the digestion procedure described in Section 3.7. Aliquots of 1 mL of the hydrolyzed solution were taken at 10, 20, 30, 60, 90, 120, and 180 min intervals, respectively. They were mixed with 4 mL of absolute ethanol to deactivate the enzymes. The glucose content of the hydrolysates was determined using the GOPOD method. The values were expressed as mg glucose/g sample. Glucose content was plotted as a function of time and the areas under the hydrolysis curves (AUC) were calculated. The hydrolysis index (HI) for each sample was calculated as the ratio between the AUC of the sample and the AUC for the reference food (white bread). The HI values were normalized for the total carbohydrate available in each sample and reference and expressed as percentages. The GI was predicted according to the equation described by Goñi, Garcia-Alonso, and Saura-Calixto [56]:

(6) GI (%) = 39.71 + 0.549×HI

### 3.10. Statistical Analysis

All assays were performed in triplicate. All data are presented as means plus standard deviation. Statistical analyses were carried out using Statistica (version 13.0, StatSoft, Krakow, Poland). Tukey’s test was used to compare the groups. The differences between the mean values were found to be statistically significant at a *p* values of less than 0.05.

## 4. Conclusions

This study was undertaken to identify the potential for edible insects to increase the nutritional and antioxidant properties of shortcake biscuits. The results revealed that the substitution of wheat flour with mealworm flour changed the nutritional value of the products. There was a progressive increase in the protein and ash contents of biscuits as the concentration of mealworm flour increased. In turn, the fat content and energy value did not differ significantly.

Increased levels of mealworm flour in the biscuits led to a darker appearance. Additionally, the biscuit with the greatest amount of mealworm flour was characterized by the highest spread factor. Regarding the antioxidant activity, mealworms were found to have high antioxidant potential, as evident from the higher free radical scavenging activity of biscuits enriched in mealworm flour in comparison to control. Moreover, it is very beneficial that the addition of mealworm flour to biscuits caused an increase in slowly digested starch, with a decrease in rapidly digested starch.

The results showed that edible insects might be a good nutritive and bioactive additive to traditionally eaten food, which was confirmed based on fortification of the shortcake dough. This type of dough can be used as an intermediate product to make various types of food, including not only confectionery products, but also savory products. A further step would be to investigate the effects of the addition of edible insects to other food matrices.

## Figures and Tables

**Table 1:** Physical properties of the biscuits.

Samples	Weight (g)	Diameter (cm)	Thickness (mm)	Spread Ratio	Apparent Density (g/cm^3^)
C	12.37 ± 0.92 ^b^	4.98 ± 0.06 ^a^	8.04 ± 0.5 ^a^	6.19 ± 0.3 ^b^	0.197 ± 0.01 ^b^
M1	10.88 ± 0.82 ^c^	4.94 ± 0.05 ^a^	6.89 ± 0.47 ^b^	7.18 ± 0.5 ^a^	0.204 ± 0.01 ^a^
M2	13.41 ± 0.94 ^a^	4.97 ± 0.07 ^a^	8.33 ± 0.32 ^a^	5.96 ± 0.2 ^b^	0.207 ± 0.01 ^a^
M3	12.39 ± 0.9 ^b^	4.96 ± 0.05 ^a^	8.11 ± 0.31 ^a^	6.12 ± 0.2 ^b^	0.196 ± 0.02 ^b^

C—control sample; M1—modification 1; M2—modification 2; M3—modification 3. Mean values with different letters in each column are significantly (*p* < 0.05) different.

**Table 2:** Nutritional values of the biscuits.

Sample	Protein (% d.w.)	Fat (% d.w.)	Ash (% d.w.)	Carbohydrates (% d.w.)	Moisture (%)	Energy Value (kcal/100g d.w.)	Energy Value (kJ/100g d.w.)
C	9.09 ± 0.46 ^c^	27.03 ± 1.48 ^a^	0.28 ± 0.04 ^b^	63.6 ± 1.63 ^a^	6.4 ± 0.23 ^a^	534 ± 4.3 ^a^	2236 ± 12.8 ^a^
M1	13.52 ± 0.6 ^a^	27.17 ± 0.39 ^a^	0.63 ± 0.06 ^a^	58.69 ± 1.85 ^b^	4.33 ± 0.09 ^c^	533 ± 4.5 ^a^	2233 ± 13.3 ^a^
M2	11.97 ± 0.5 ^b^	26.97 ± 1.69 ^a^	0.7 ± 0.1 ^a^	60.36 ± 1.49 ^ab^	5.3 ± 0.16 ^b^	532 ± 4.9 ^a^	2227 ± 12 ^a^
M3	10.82 ± 0.5 ^b^	28.47 ± 0.36 ^a^	0.44 ± 0.05 ^b^	60.27 ± 1.72 ^ab^	5.97 ± 0.2 ^a^	541 ± 3.9 ^a^	2262 ± 12.7 ^a^

C—control sample; M1—modification 1; M2—modification 2; M3—modification 3, Values followed by a different superscript in a column differ significantly (*p* < 0.05).

**Table 3:** Color determinants of biscuits.

Sample	L*	a*	b*	∆E	BI
C	34.44 ± 2.97 ^a^	5.77 ± 1.63 ^b^	8.61 ± 0.94 ^a^	-	11.80
M1	20.96 ± 0.96 ^d^	8.64 ± 1.31 ^a^	2.14 ± 0.64 ^c^	15.23	26.67
M2	26.45 ± 0.56 ^c^	6.57 ± 1.01 ^ab^	5.62 ± 0.81 ^b^	8.56	17.18
M3	29.46 ± 0.97 ^b^	7.12 ± 0.8 ^ab^	7.46 ± 0.3 ^ab^	5.28	16.75

C—control sample; M1—modification 1; M2—modification 2; M3—modification 3. Values followed by a different superscript in a column differ significantly (*p* < 0.05). * mean in terms of lightness (L*) and color (a*—redness; b*—yellowness).

**Table 4:** Rapidly and slowly digested starch contents and in vitro glycemic index values for the biscuits.

Sample	RDS (mg Glucose/g Sample)	SDS (mg Glucose/g Sample)	In Vitro GI
C	223.28 ± 9.85 ^a^	73.55 ± 2.69 ^b^	39.85 ± 0.12 ^a^
M1	181.66 ± 9.61 ^b^	102.95 ± 6.12 ^a^	39.83 ± 0.1 ^a^
M2	191.8 ± 5.58 ^b^	91.01 ± 6.75 ^a^	39.84 ± 0.08 ^a^
M3	218.52 ± 1.85 ^a^	72.10 ± 4.89 ^b^	39.85 ± 0.15 ^a^

C—control sample; M1—modification 1; M2—modification 2; M3—modification 3; RDS—rapidly digested starch; SDS—slowly digested starch. Values followed by a different superscript in a column differ significantly (*p* < 0.05).

**Table 5:** Recipe for shortcake biscuits.

Ingredients	C	M1	M2	M3
Wheat flour (g)	300	270	280	285
Butter (g)	150	135	140	142.5
Sugar (g)	70	70	70	70
Eggs (pcs)	1	1	1	1
Mealworm flour (g)	-	30	20	15

C—control sample; M1—modification method 1; M2—modification method 2; M3—modification method 3.
